# The Influence of Different Rope Jumping Methods on Adolescents’ Lower Limb Biomechanics during the Ground-Contact Phase

**DOI:** 10.3390/children9050721

**Published:** 2022-05-13

**Authors:** Yi Lin, Zhenghui Lu, Xuanzhen Cen, Anand Thirupathi, Dong Sun, Yaodong Gu

**Affiliations:** 1 Faculty of Sports Science, Ningbo University, Ningbo 315211, China; linyi_nbu@hotmail.com (Y.L.); luzhenghui_nbu@foxmail.com (Z.L.); cenxuanzhen@outlook.com (X.C.); ananthzeal@gmail.com (A.T.); 2 Faculty of Engineering, University of Szeged, H9700 Szeged, Hungary

**Keywords:** rope jumping, adolescent, biomechanics, electromyography

## Abstract

As a simple and beneficial way of exercise, rope skipping is favored by the majority of teenagers, but incorrect rope skipping may lead to the risk of injury. In this study, 16 male adolescent subjects were tested for bounced jump skipping and alternating jump rope skipping. The kinematic data of the hip, knee, ankle and metatarsophalangeal joint of lower extremities and the kinetics data of lower extremity touching the ground during rope skipping were collected, respectively. Moreover, the electromyography (EMG) data of multiple muscles of the lower extremity were collected by Delsys wireless surface EMG tester. Results revealed that bounced jump (BJ) depicted a significantly smaller vertical ground reaction force (VGRF) than alternate jump (AJ) during the 11–82% of the ground-contact stage (*p* < 0.001), and the peak ground reaction force and average loading rate were significantly smaller than AJ. From the kinematic perspective, in the sagittal plane, when using BJ, the flexion angle of the hip joint was comparably larger at 12–76% of the ground-contact stage (*p* < 0.01) and the flexion angle of the knee joint was significantly larger at 13–72% of the ground-contact stage (*p* < 0.001). When using two rope skipping methods, the minimum dorsal extension angle of the metatarsophalangeal joint was more than 25°, and the maximum was even higher than 50°. In the frontal plane, when using AJ, the valgus angle of the knee joint was significantly larger during the whole ground-contact stage (*p* < 0.001), and the adduction angle of the metatarsophalangeal joint (MPJ) was significantly larger at 0–97% of the ground-contact stage (*p* = 0.001). EMG data showed that the standardized value of root mean square amplitude of the tibialis anterior and gastrocnemius lateral head of BJ was significantly higher than AJ. At the same time, that of semitendinosus and iliopsoas muscle was significantly lower. According to the above results, compared with AJ, teenagers receive less GRF and have a better landing buffer strategy to reduce load, and have less risk of injury during BJ. In addition, in BJ rope skipping, the lower limbs are more inclined to the calf muscle group force, while AJ is more inclined to the thigh muscle group force. We also found that in using two ways of rope skipping, the extreme metatarsophalangeal joint back extension angle could be a potential risk of injury for rope skipping.

## 1. Introduction

As a convenient, low-cost and beneficial sport, rope skipping is widely welcomed by people of all ages worldwide. Under the current COVID-19 pandemic [1], rope skipping as an alternative aerobic exercise that can be carried out in a small space is becoming increasingly accepted by people [2,3]. Most studies have shown that rope skipping helps to enhance the human body’s cardiopulmonary function, muscle strength, coordination, endurance [4], agility [5], bone health [6,7] and balance [8]. Rope skipping is particularly popular among teenagers. Nearly 1 million students from 4000 schools in Canada participate in rope skipping every year [9]. In most schools in Asia, rope skipping is included in students’ sports activities [10]. Teenagers are in a critical period of physical development, a period of rapid development of body shape, function and quality. This critical period should be valued to promote their healthy growth [11]. As a good way, rope skipping plays a positive role in teenagers’ physical and mental development. Yang et al. [12] found that 12 weeks of rope skipping exercise intervention can significantly improve adolescents’ muscle strength and flexibility. Eler et al. [5] also found that ten weeks of rope skipping training positively affected teenagers’ body weight, fat ratio, speed, leg strength and maximum oxygen uptake. Despite the many benefits of rope skipping to teenagers, incorrect rope skipping movements or unreasonable content arrangements may also cause some damage.

There are many kinds of rope skipping, but their common feature is repeating continuous vertical jumps at a low height. BJ and AJ are two of the most common ways people jump rope. BJ refers to jumping and landing with both feet at the same time, which is the simplest technique of rope skipping. AJ refers to jumping with a single foot alternatively and landing with the other foot, i.e., jumping with the left foot and landing with the right foot, and then jumping with the right foot and landing with the left foot. The jumping movement can be divided into three stages: flight, landing and take-off [8]. When the foot touches the ground at the end of the take-off stage, the ground reaction force will impact the human body. To prevent injury during the landing buffer stage of the jump movement, the human body first completes this response by adjusting the stiffness of the lower limbs, i.e., by increasing the vertical displacement of the knee joint and hip joint. Aizawa et al. [13] found that increasing the knee and hip flexion angle may reduce the impact force during the landing mission. Another research [14] showed that lower limb joints have a wide range of motion (ROM) in the sagittal plane, providing sufficient shock absorption to reduce the load on fragile soft tissue structures such as ligaments. Moreover, different ways of jumping and landing showed different energy dissipation on each joint. In the process of high-frequency rope skipping, due to the high peak value of vertical ground reaction force (VGRF), passive impulse and joint load rate, the range of joint flexion of lower extremities as a buffer tool is also small, which may increase the hidden risk of lower limb injury in the long run [15]. It has been hypothesized that the development of overuse injuries, though multifactorial and variable between specific injuries, is associated with repetitive loading and insufficient recovery time [16]. Repetitive loading, especially at a high magnitude, accumulates microdamage in both tendon and bone [17]. Without adequate repair, damage accumulation may eventually lead to injury (e.g., tendinopathy or stress fracture). Tendinopathies, medial tibial stress syndrome and stress fractures have all been reported in competitive jump rope. The biomechanical analysis method can be used to analyze rope skipping movements more scientifically and reasonably. Tian et al. [18] reported the biomechanical characteristics of single-swing and multi-swing rope skipping movements through this method. Bruce et al. [2] also studied the biomechanical characteristics of single-swing rope skipping, double-swing rope skipping and running. Moreover, Pittenger et al. [19] compared the VGRF of children during the bounced jump (BJ) and alternating jump (AJ) rope skipping. Additionally, results showed that the average value of AJ’s maximum VGRF is significantly higher than that of BJ. However, in the study of biomechanical characteristics of BJ and AJ, Chow et al. [20] found that the knee flexion angle of AJ is larger than that of BJ at the initial contact with the ground. In contrast, the peak value of GRF is relatively lower, and it is considered that AJ may have a lower risk of injury than BJ. However, the above conclusion is not consistent with the results observed by Pittenger et al. (i.e., the GRF is greater than that of BJ when AJ). In the study by Chow et al. [21], the knee joint kinematic in the sagittal plane during BJ and AJ were analyzed, but this led to some conditions of the lower limb joints in the frontal plane which cannot be reflected during rope skipping. Regarding kinetics, the previous studies about rope skipping only focus on the analysis of the peak GRF data. Additionally, these studies assumed that the time of the peak point of GRF is consistent with the critical time of damage, which may ignore the important time fluctuations that may occur in the whole ground-contact phase. Statistical parametric mapping (SPM) is a method to test the statistical differences of continuous data such as kinematics and kinetics over the whole period of motion, and to accurately calculate the significance threshold [22,23,24,25]. The differences in sports biomechanical characteristics between the two rope skipping methods can be better revealed by statistically analyzing the biomechanical data based on traditional discrete analysis and one-dimensional statistical parametric mapping (spm1d). As the majority of participants in rope skipping, teenagers should have a scientific understanding of rope skipping technology to avoid the risk of injury and promote physical and mental development [19]. Therefore, to further explore the differences in the biomechanical characteristics of lower limbs during the ground-contact phase in different ways, and to judge the risk of injury in rope skipping sports to provide the theoretical basis for the prevention of related injuries and suggestions for the scientific development of rope skipping, we (a) analyze the differences in the kinematic characteristics of the hip, knee, ankle and the metatarsophalangeal joint between the two rope skipping methods in the sagittal and frontal plane, (b) analyze the kinetics of the two rope skipping methods and (c) collect electromyography (EMG) data of leg muscle activation and fatigue during rope skipping. We assume that: (a) the angle change in the joint during BJ has a better buffering strategy than AJ, (b) the GRF of BJ is smaller than AJ and (c) the EMG activation of the calf muscle when performing BJ is stronger than AJ.

## 2. Materials and Methods

### 2.1. Participants

A total of 16 male subjects (age: 13.2 ± 0.65 years old, height: 169.18 ± 3.67 cm, weight: 54.98 ± 5.60 kg) were recruited. The subjects were required to have more than one year of training experience and be proficient in BJ and AJ. We only measured data on the dominant leg, which is defined as the leg used for playing soccer, and for all our subjects, their dominant legs are the right leg. All the subjects were in good physical condition and exercise ability. There were no lower limb sports injuries, no musculoskeletal system and other related diseases in the past six months. This study was approved by the Human Ethics Committee in the Research Institute of Ningbo University (ARGH20211115).

Before the experiment, the parents or relevant guardians of the subjects understood the research purpose, experimental requirements and process. They signed the informed consent form of the experimental subjects. The ethics committee of the university approved the study. The specific information is shown in Table 1 below.

### 2.2. Experimental Apparatus

**Figure 1:**
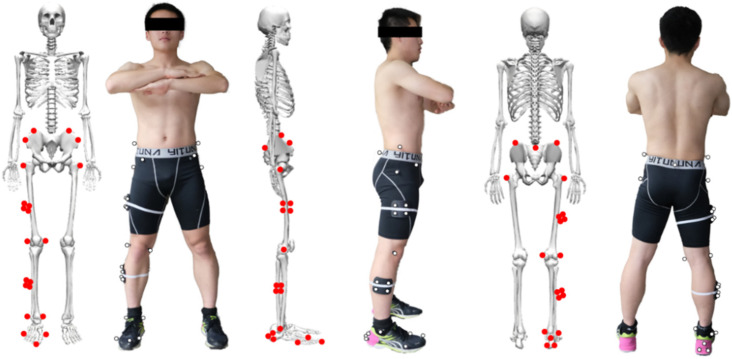
The placement of the reflective markers (front view, side view and back view) following bony anatomical landmarks.

Vicon 3D capture system (Oxford Metrics Ltd., Oxford, UK): In this study, the infrared 3D motion capture system produced by Vicon Company includes eight infrared cameras. Moreover, 25 mark points were pasted to define the pelvis, thigh, shank, forefoot and hindfoot, which were glued to the subject’s sacroiliac joint center (SACR), left and right anterior superior iliac spine (LASI, RASI), left and right iliac crest (LPP, RPP), left and right greater trochanter (LTROC, RTROC), right thigh tracking point (S1, S2, S3, S4), right inner and outer knee (RMK, RLK), right calf tracking point (ST1, ST2, ST3, ST4), right inner and outer ankle (RMA, RLA), right leg heel tracking points (SH1, SH2, SH3), the right big toe (RTOE), the first metatarsophalangeal joint (RM1) and the fifth metatarsophalangeal joint (RM5) (Figure 1) [26,27]. According to the basic requirements of the three-dimensional reconstruction, these mark points (diameter: 9.5 mm) were used to accurately stick to the bony landmarks of each part of the subject’s body. Eight infrared motion capture cameras were used to simultaneously capture the motion trajectory of mark points, thus generating the movement process of the human body model. The changes in the joint angle parameters in the sagittal and frontal plane during the jump rope ground-contact phase were collected.

AMTI force platform (AMTI, Watertown, MA, USA): The force platform was used to measure the change in the GRF in the front, back, left and right and vertical directions of the human body during the process of skipping rope in two different ways. In this experiment, an AMTI force platform (40 cm × 60 cm) was embedded in the floor to complete kinetic data acquisition with 1000 Hz of acquisition frequency. The force platform was connected to the computer, which can realize the synchronous acquisition with the Vicon infrared capture system. The data processing terminal is Vicon Nexus 1.8.1 (Vicon Motion System Ltd., Oxford, UK).

Delsys wireless surface EMG tester (Delsys, Boston, MA, USA) was used to collect EMG data from the right lower gastrocnemius medial head, gastrocnemius lateral head, tibialis anterior, rectus femoris, semitendinosus and iliopsoas muscle.

### 2.3. Experimental Protocol and Procedures

**Figure 2:**
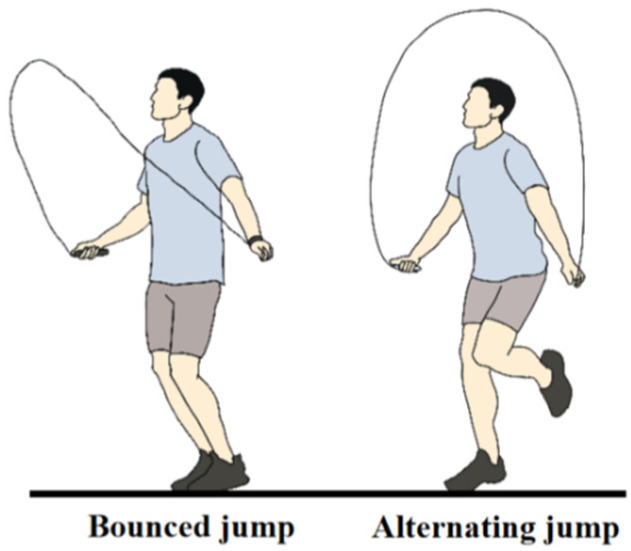
A diagram of bounced jump (BJ) and alternating jump (AJ).

Before the experiment, each subject wore the experimental sneakers and performed a series of warm-up activities, including dynamic stretching and small load exercises for the upper, lower limbs and core, and used the uniformly designated skipping rope (the length of the rope according to the subject’s suitable length). The warm-up was performed with two kinds of skipping ropes so that the subjects could adapt to the experimental environment, skipping rope and shoes. After the warm-up, all subjects were required to wear tight pants, and mark points were pasted on the specified body positions according to the requirements of the model. After the static motion capture experiment, the subjects chose a comfortable skipping frequency for the test, then performed BJ and AJ. The order of two skipping ropes was random during the test. A schematic diagram of two kinds of rope skipping is shown in Figure 2. Their right foot completely fell on the force platform as a successful acquisition. The subjects continuously skipped rope more than ten times, and the successful middle one was taken as the analysis object. For each subject, ten successful data needed to be collected. There was 1 min before and after each rope skipping collection to ensure the subject’s full recovery during each test. At the same time, the AMTI force measuring platform was used to collect the changes in parameters such as peak vertical ground reaction force (VGRF) and vertical impact loading rate (VALR) during rope skipping. Delsys wireless surface EMG tester was used to collect the muscle EMG, including internal gastrocnemius medial head, gastrocnemius lateral head, tibialis anterior, rectus femoris, semitendinosus and iliopsoas during rope skipping. While collecting EMG data, the EMG sensors were stuck to the most prominent part of the six muscles and a razor was used to remove surface hair, body surface grease, sweat and other foreign bodies that may affect EMG signal collection [28]. The EMG signals of the subjects during rope skipping were measured synchronously, and the degree of muscle activation and fatigue were judged by EMG data to indirectly reflect the different characteristics between the two rope skipping methods. In order to standardize the EMG data, we also collected the surface EMG signals of the maximum voluntary isometric contraction (MVC) contraction of the above muscle activities. The MVC test time of each muscle lasted more than 5 s, reached the maximum value in the first 2 s and maintained the maximum strength for 3 s. Each muscle was tested three times, and at least 5 min intervals were taken to eliminate fatigue [29,30,31].

### 2.4. Data Collection and Processing

The three-dimensional kinematics data of hip, knee, ankle and metatarsophalangeal joint and GRF data were processed and analyzed from the moment when rope skipping touched the ground to the end of leaving the ground. The joint angle–time curve and the ground reaction–time curve were standardized as a percentage of the action cycle through the action cycle (0–100%). The GRF was standardized by the subject’s body weight (BW), and the effective data of each parameter were averaged. The collected EMG data were filtered and smoothed by EMG works Analysis software (Delsys, Boston, MA, USA). Finally, the root mean square amplitude was calculated. The specific steps of EMG data post-processing are as follows: (1) Use EMG Works Analysis software to open sampled experimental data; (2) Apply to remove mean operation to the EMG data; (3) Apply band-pass filtering to filter signals less than 10 Hz and greater than 500 Hz by using the Filter IIR tool; (4) Calculate the absolute value of EMG data in simple math for full-wave rectification; (5) Finally, use root mean square tool to calculate the RMS of EMG signal in the ground-contact phase. Because the surface EMG signal index has individual differences and is affected by different muscles, it is not suitable to use the measured values of surface EMG to compare and analyze directly. It is necessary to standardize the time domain index in time-domain analysis. The method of standardized processing was to take the surface EMG signal of the target muscle during the maximum isometric contraction as the denominator and the surface EMG signal of the muscle activity as the numerator to calculate the ratio of them and express it as a percentage (Equation (1)) [32,33].

(1) $$\mathrm{Root}\;\mathrm{mean}\;\mathrm{square}\;\mathrm{amplitude}\;\mathrm{normalized}\;\mathrm{value}=\frac{\mathrm{root}\;\mathrm{mean}\;\mathrm{square}\;\mathrm{amplitude}\times \;100\%\;}{\mathrm{root}\;\mathrm{mean}\;\mathrm{square}\;\mathrm{amplitude}\;\mathrm{of}\;\mathrm{MVC}}$$

### 2.5. Statistical Analysis

IBM SPSS Statistics19 software (SPSS Inc, Chicago, IL, USA) was used for statistical analysis. The normality of the data was verified by the Shapiro–Wilk test. The differences between AJ and BJ were analyzed by the paired sample t-test of lower limb kinematics, kinetics and EMG root mean square amplitude normalization. For SPM 1d analysis, each task generated a separate curve before analysis. Then, all data were extracted to expand into time series curves with 101 points using a custom MATLAB script. The 101 data points represent the ground-contact phase (0–100%). Statistical analysis was performed using the open-source script of SPM1d (paired samples t-test), and the significance level was set at 0.05 [34].

## 3. Results

**Figure 3:**
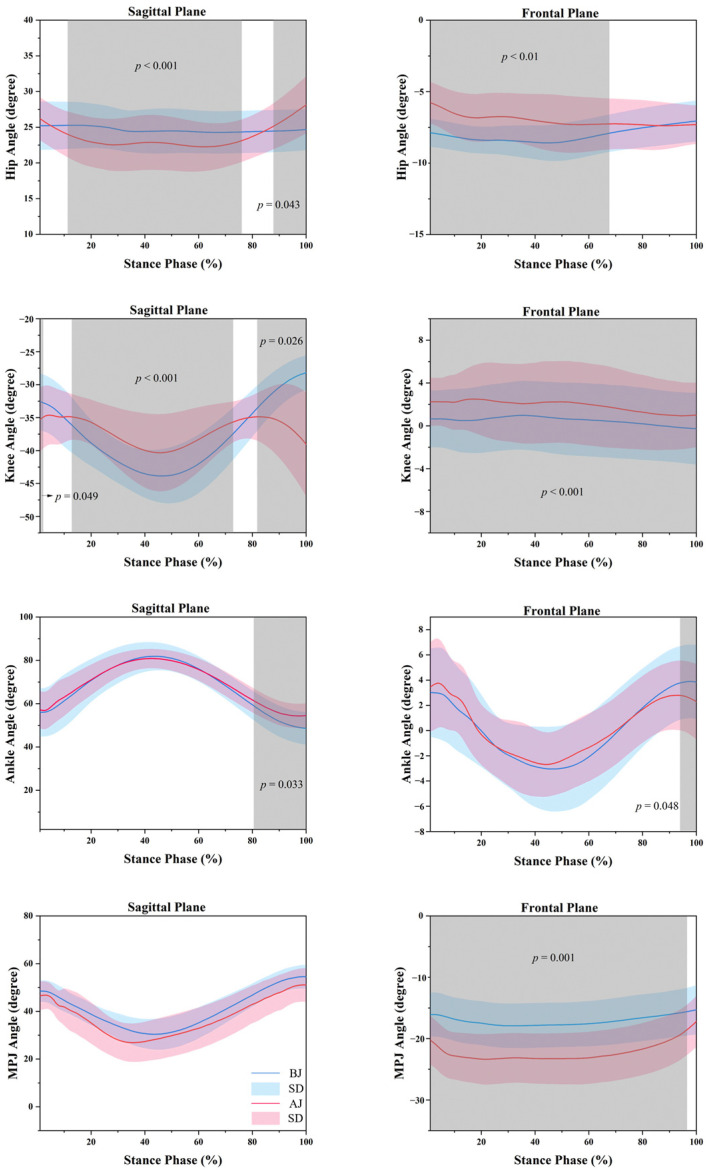
The statistical parametric mapping (SPM) results between bounced jump (BJ) and alternate jump (AJ) tasks following a spike, depicting the mean angle and standard deviation (SD) of the ankle, knee, hip and metatarsophalangeal joint sagittal and frontal plane. Grey shaded areas indicate significant differences between BJ and AJ during the ground-contact phase (*p* < 0.05).

**Figure 4:**
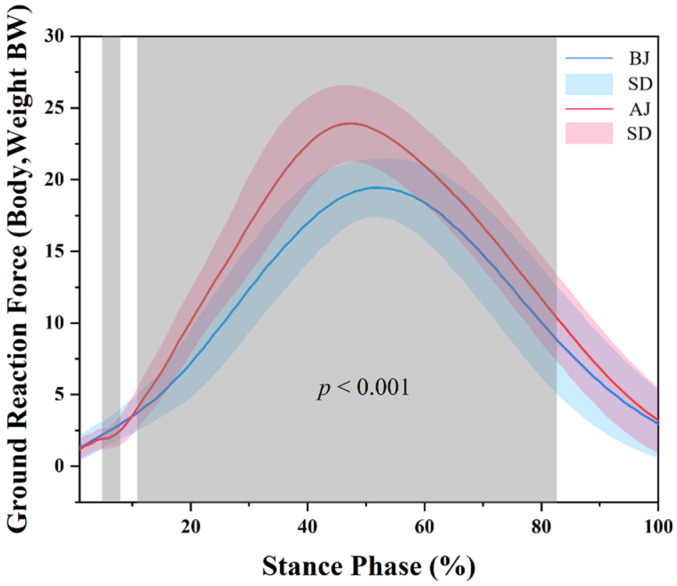
The statistical parametric mapping (SPM) results between bounced jump (BJ) and alternate jump (AJ) tasks following a spike, depicting the mean vertical ground reaction force (VGRF) and standard deviation. Grey shaded areas indicate significant differences between BJ and AJ during the ground-contact phase (*p* < 0.05).

**Figure 5:**
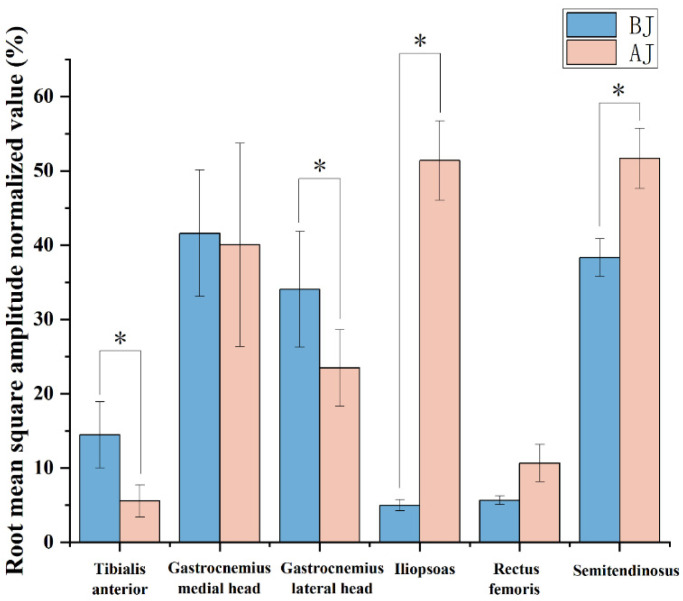
The mean electromyography (EMG) data of lower limb muscles through the ground-contact phase. Note: * represents a significant difference between bounced jump (BJ) and alternate jump (AJ), *p* < 0.05.

If the *p*-value of the Shapiro–Wilk test is greater than 0.05, then the data have a normal distribution. The angle of joint, GRF and the average loading rate of GRF and EMG data can be seen in Figure 3, Figure 4 and Figure 5, Table 2.

### 3.1. Kinematics

The results of hip, knee, ankle and metatarsophalangeal joint angle of foot landing in different ways of rope skipping are shown in Figure 3.

For the results of the hip joint, SPM analysis revealed that in the sagittal plane, BJ had a significantly greater flexion angle than AJ during the 12–76% (*p* < 0.001) and a significantly smaller flexion angle than AJ during the 88–100% (*p* = 0.043). In the frontal plane, BJ depicted a significantly greater adduction angle than AJ during the 0–68% (*p* < 0.01). For the results of the knee joint, SPM analysis revealed that in the sagittal plane, BJ had a significantly smaller flexion angle than AJ during the 0–2% (*p* = 0.049), a significantly greater flexion angle than AJ during the 13–72% (*p* < 0.001) and a significantly smaller flexion angle than AJ during the 82–100% (*p* = 0.026). In the frontal plane, BJ depicted a significantly smaller valgus angle of the knee joint than AJ during the 0–100% (*p* < 0.001). SPM analysis revealed that in the sagittal plane, BJ had a significantly smaller dorsiflexion angle than AJ during 81–100% (*p* = 0.033) for the ankle joint. In the frontal plane, BJ depicted a significantly greater valgus angle than AJ during the 94–100% (*p* = 0.048). For the results of the metatarsophalangeal joint, SPM analysis revealed no significant difference in the sagittal plane between BJ and AJ during rope skipping. In the frontal plane, BJ depicted a significantly smaller adduction angle than AJ during the 0–97% (*p* = 0.001). 

### 3.2. Kinetics

The results of kinetics are shown in Figure 4. SPM analysis revealed that BJ had a significantly greater VGRF than AJ during the 4–7% (*p* = 0.047) and a significantly smaller VGRF than AJ during the 11–82% (*p* < 0.001).

The peak GRF of BJ was 20.24 ± 1.89 BW, and that of AJ was 24.91 ± 2.71 BW. The peak GRF of BJ was significantly smaller than that of AJ in the ground-contact phase when skipping rope was performed (*p* = 0.001). The average loading rate of BJ was 365.68 ± 51.40 BW/s, and that of AJ was 510.73 ± 94.02 BW/s. The average loading rate of BJ was significantly lower than that of AJ in the landing stage when jumping rope was performed (*p* = 0.001).

### 3.3. Surface EMG

During the whole lower limb ground-contact phase of rope skipping, the root mean square amplitude normalization value and standard deviation of each muscle of lower extremities with different rope skipping methods are shown in Table 2 and Figure 5. The results showed that the standardized value of EMG RMS amplitude of tibialis anterior during BJ was 14.46 ± 4.45%, and AJ was 5.57 ± 2.15%, which was significantly higher than that of AJ (*p* = 0.001). The standardized value of EMG RMS amplitude of gastrocnemius lateral head during BJ was 34.05 ± 7.79%, and AJ was 23.49 ± 5.14%, significantly higher than AJ (*p* = 0.026). The normalized value of root mean square amplitude of iliopsoas during BJ was 5 ± 0.756%, and AJ was 51.38 ± 5.317%, which was significantly lower than AJ (*p* = 0.001). The standardized value of the root mean square amplitude of semitendinosus during BJ was 38.33 ± 2.52%, and AJ was 51.67 ± 4.04%, significantly lower than AJ (*p* = 0.015). There was no significant difference between BJ and AJ in the EMG results of the other two muscles.

## 4. Discussion

The kinetic results showed that AJ’s peak VGRF and average loading rate were significantly greater than BJ when skipping rope. This result is consistent with our initial hypothesis. The SPM1d results showed that the GRF of AJ was significantly greater than that of BJ at 11–82% of the ground-contact stage. This result is consistent with the study carried out by Pittenger et al. [19], nevertheless, partially inconsistent with the study by Chow et al. [20]. The knee flexion angle of the two skipping positions was the largest at 46% of the ground-contact phase, while the GRF index showed that the BJ peaked at 51% of the ground-contact phase and AJ reached the peak at 47% of the ground-contact phase. The GRF did not peak at the maximum knee flexion angle but peaked at the extension after maximum flexion. The lower limb landing stage of a continuous vertical jump, such as skipping rope, differed from the general jump landing. Immediately after knee flexion and cushioning, the lower extremity needs to be stirred off the ground [35], which means that, in addition to GRF, the lower extremity receives additional reaction forces caused by active kicking during landing. Thus, it is considered that the peak point of the GRF is the initial kicking and extension immediately after the flexion of the lower limb reaches the maximum.

A high-impact landing will place a greater load on the lower limbs, increasing the probability of injury. A good landing posture is not only a manifestation of good athletic skills but also an effective means of preventing lower extremity injuries. During landing, people usually slow down the impact on themselves by muscle contraction strategies and adjusting joint angles to reduce the load on the lower extremities [35,36]. The hip flexion angle during BJ was significantly larger than AJ at 12–76% of the ground-contact stage, and BJ might be able to reduce loads to a certain extent through a larger hip flexion angle. The hip flexion angle of AJ was significantly greater than that of BJ during 88–100% of the ground-contact phase. Combined with EMG data, it is found that when BJ skipped rope, the calf muscle group EMG activation level was higher than that of AJ, and the activation of tibialis anterior and gastrocnemius lateral head was significantly higher than that of AJ. The muscle EMG activation level of the thigh muscle group was higher than that of BJ when performing rope skipping in AJ, and the EMG activation level of iliopsoas and semitendinosus of AJ was significantly higher than that of BJ. Therefore, we speculate that AJ mainly uses the active contraction of the iliopsoas and semitendinosus to flex the hips and knees to lift the leg off the ground in the stage of kicking off the ground at the later stage of the jump rope touchdown. In contrast, the BJ mainly uses the tricep calf. Additionally, agonistic muscle contraction of the ankle causes plantar flexion of the ankle to lift off the ground.

This hypothesis is consistent with the results of the EMG tests in the experiment, and the AJ’s greater hip flexion angle at 88–100% of the ground-contact phase can be attributed to the active hip flexion caused by its iliopsoas contraction. While in Figure 3, it can be observed that AJ first performed the extension and depression of the hip joint before the time of the maximum peak GRF (i.e., before the maximum flexion stage of the knee joint) and then performed the hip flexion and leg lift during the stage of pushing off and leaving the ground. This indicates that the greater GRF of AJ jump rope landing may be related to the impact force brought by the active extension of the hip joint during the initial landing [21]. Similarly, the knee flexion angle of BJ was significantly greater than that of AJ at 13–72% of the ground-contact phase, which may reduce the load on the lower limbs. At 82–100%, due to the contraction of the biceps femoris muscle at the back of the thigh, AJ needs to lift the leg, causing the knee to lift the leg off the ground. At the same time, the BJ is more reflected in the knee stretch to make the human body jump into the air, so the flexion angle of the knee joint is significantly greater than that of the BJ jump rope during the pedal and stretch period. The ankle joint is passively transformed from plantar flexion to dorsiflexion when landing. Then, when the plantar flexors contract to cause plantar flexion to push the ground, a force is exerted on the ground to provide power for the next jump. 

Before the feet touch the ground when landing, to reduce the impact force exerting on the lower limbs, people can use the energy absorption capability of the plantar flexor muscles of the ankle joint by adequately increasing the plantarflexion angle [37]. The dorsiflexion angle of AJ was significantly larger than that of BJ during the 81–100%. It can be seen from the curve that the BJ tends to plantarflexion during the pedal and stretch period, which may provide energy absorption for the next BJ landing on the ankle. Furthermore, the EMG results in greater muscle activation in the calf muscles at the BJ also provided solid evidence for this hypothesis. No significant difference in the metatarsophalangeal joint angle was found between AJ and BJ on the sagittal plane. We found that the metatarsophalangeal joints passively dorsi-extended when they first touched the ground. With the gradual increase in GRF after landing, the dorsiflexion angle of the metatarsophalangeal joints gradually became smaller. This could be an energy absorption strategy of the human body by changing the lower extremity joint angle to reduce stress on the metatarsophalangeal joint.

Li et al. [38] studied the metatarsal stress under different landing angles of the forefoot by the finite element method. They found that the bending strain energy produced in the long axis direction and the shear strain energy produced in the cross-section were larger. The soft tissue surrounding the metatarsal will also undergo greater deformation, and these high-strain tissues create high stresses within the metatarsal. This increases the internal energy in the foot system during the landing of the forefoot, which significantly increases the probability of metatarsal fractures. In addition, when the forefoot touches the ground at an angle of 15° or more, the metatarsal stress is significantly increased, and the change in stress distribution on the bone and the high cycle load are important causes of stress fractures in sports. In this study, we found that the minimum dorsiflexion angle of the metatarsophalangeal joint was greater than 25° and the maximum even reached more than 50°, which may bring a high load to the metatarsal bones. The cyclic stress greatly increases the probability of injury, and excessive dorsiflexion of the metatarsophalangeal joint is also likely to cause injury diseases such as turf-toe [39,40]. 

By examining the kinematics results in the frontal plane, during the ground-contact phase, it was observed that BJ had a more adducted hip joint angle compared to AJ, while the knee joint valgus angle and metatarsophalangeal joint adducting angle of AJ were significantly greater than those of BJ almost throughout the entire stage. BJ had more adduction of the hip joint angle than AJ, and two reasons may cause this phenomenon. Firstly, the action of BJ puts legs close together, which will cause a slightly larger hip adducted angle. Secondly, it may be caused by the larger hip flexion angle when landing. When the hip is flexed, the hip joint will be wider in the frontal plane than the knee joint, which will lead to a larger flexion angle in the sagittal plane, and the adduction angle in the frontal plane also increases [41]. 

The valgus angle AJ of the knee joint was significantly larger than that of BJ during the whole ground-contact phase, and the GRF of AJ was significantly larger than that of BJ, with the load being carried by one leg during AJ. Moreover, the knee flexion angle in the sagittal plane was smaller than that of BJ, and the knee joint of AJ bears a greater load than BJ, with the landing cushioning worse than BJ. Moreover, the larger knee valgus angle may interfere with the gravitational line of the lower extremity joints to cause instability, and it increases the load on the medial ligaments of the knee joint and the pressure on the lateral articular cartilage [42], which may lead to a greater probability of knee injury. The metatarsophalangeal adduction angle was significantly greater during AJ than BJ. However, there was no significant difference in the sagittal plane of the metatarsophalangeal joint in the two jumping positions. Based on the previously mentioned greater dorsiflexion of this joint, AJ had greater adduction than BJ. In addition, a more significant GRF will greatly increase the probability of metatarsophalangeal joint injuries and diseases such as hallux valgus [43].

To summarize, the energy absorption brought by the sagittal hip, knee and ankle flexion buffer strategy during BJ skipping for adolescents is better than that of AJ. AJ had a greater knee joint valgus angle and metatarsophalangeal joint adduction angle in the frontal plane compared to BJ. Combined with the kinetic results of AJ, this may increase the probability of injury in adolescents skipping rope. Adolescents who use AJ skipping rope may have a greater risk of lower extremity damage than during BJ, which is inconsistent with the conclusion from Chow et al. which that BJ had a lower risk of injury than AJ. However, the inconsistency may be related to the age of the subjects. The average age of male subjects in the study of Chow et al. was 23.5, while the subjects in our study were adolescents with an average age of 13.2. Adults have a greater body mass than adolescents, which generates a greater GRF upon landing [44]. However, the lower extremity muscles of adults are more developed than those of adolescents, and the control strategy of muscle contraction and bone angle when landing is more prominent than adolescents [45]. The greater flexion angle of the knee joint could also be explained when landing with a single leg in Chow’s study. Moreover, the EMG results reflected that BJ tends to use calf muscles and AJ tends to use thigh muscles, and the calf muscles have a smaller cross-sectional area than the thigh muscles [46] and are more prone to fatigue. When the BJ lands, the heavier weight of the adult is cushioned by the eccentric contraction of the posterior calf muscle group. Compared with the eccentric contraction of the AJ front thigh muscle group, it may be challenging to achieve the muscle contraction and cushioning of the AJ, which may be the reason for the higher injury risk of BJ for adults than that of AJ.

There are many soft tissues in the skeletal system and plenty of water and organics in bone tissue but few inorganic salts for adolescents. Their bones have good elasticity and are difficult to break, but they are easy to bend and have poor firmness [47]. Therefore, it is necessary to scientifically guide young people to use the correct rope skipping action, choose the appropriate rope skipping method, reasonably arrange the exercise load and use exercise aids to reduce the load on the lower limbs during exercise and strengthen the protection of their bodies, especially considering that they are in a critical period of body growth and development. When skipping rope, the function of shoes is crucial. An appropriate shoe may help offset the reductions in stiffness or external force that allow the total movement to be maintained in all conditions. Some studies have shown that wearing sports shoes with greater flexion stiffness can change the kinematics of the metatarsophalangeal joints while increasing the pedaling effect of the adjacent ankle joints and can reduce the metabolic cost of the lower limb muscles to a certain extent [48,49,50], which may play an essential role in relieving the large load on the metatarsophalangeal joint during rope skipping. Studies have also shown that different shoe sole stiffness has different effects on lower limb muscle activation or vertical stiffness during rope skipping. Higher sole stiffness may lead to greater lower limb muscle activation during exercise. In comparison, lower sole stiffness will reduce the impact force on landing, but the performance of muscle force on landing or pedaling may be weakened [51,52]. Therefore, suitable skipping shoes should balance cushioning protection, subjective force and passive labor-saving. Different people have different subjective feelings about the comfort of shoes, so it is essential to choose the right sneakers for skipping. Knee and ankle braces can also provide external protection for the joints during exercise, reduce the load of the lower limbs and enhance the stability of the joints [53,54,55]. All of the above methods can reduce the probability of lower extremity injury when skipping rope, which is very important for the healthy growth of young people.

There are some limitations to be considered in this study. First, our study subjects did not include female adolescents. Previous studies have shown differences between male and female biomechanics during the ground-contact phase [37,56,57], so future studies should consider female subjects. Second, our experiment only considered some cushioning strategies of the lower limbs when skipping rope but did not consider that the human upper limb motion may impact the lower limb biomechanics. Shimokochi et al. [58] found that leaning forward while landing protects the anterior cruciate ligament (ACL) by increasing the shock absorption capacity and knee flexion angles and decreasing anterior shear force due to the knee joint compression force and quadriceps muscle activation. However, our study may neglect the motion of the upper limbs of the human body. Therefore, future research needs to further analyze the biomechanics of the human upper limb torso during rope skipping to supplement our study.

One more limitation is that we only measured kinetics, kinematics and EMG signal of the right-side lower limb of subjects, which is the dominant side of the lower limb but did not collect data on the left side. T. N. Brown’s study found [59] that limb dominance had a substantial impact on lower limb biomechanics elicited during landings. Therefore, in the future, we will also study the biomechanics of the left-side lower limb in rope jumping.

## 5. Conclusions

In conclusion, this study analyzed the biomechanical characteristics of the lower extremities of adolescents with different styles of rope skipping (BJ and AJ) during the ground-contact phase. Firstly, the lower limbs tend to exert more force on the calf muscles during BJ, while AJ prefers the thigh muscles. Secondly, the minimum value of the dorsal extension angle was greater than 25°, and the maximum reached more than 50°. The larger metatarsophalangeal dorsiflexion angle may cause high-load cyclic stress to the metatarsal during rope skipping, increasing the probability of potential injury. Finally, BJ has a better buffering strategy in the lower limb sagittal plane than AJ to reduce lower limb landing load. AJ has a larger knee valgus angle and metatarsophalangeal joint adduction angle than BJ in the frontal plane. AJ receives greater GRF than BJ during landing, leading to a greater risk of injury when using AJ rope skipping. Therefore, we recommend that teenagers use BJ when skipping rope.

## Figures and Tables

**Table 1:** The basic information of the participants.

Number	Age (Year)	Weight (kg)	Height (cm)
16	13.2 ± 0.65	54.98 ± 5.60	169.18 ± 3.67

**Table 2:** The mean electromyography (EMG) data of lower limb muscles through the ground-contact phase.

	Root Mean Square Amplitude Normalized Value %	
	BJ	AJ
Tibialis anterior	14.46 ± 4.45 *	5.57 ± 2.15
Gastrocnemius medial head	41.59 ± 8.51	40.03 ± 13.67
Gastrocnemius lateral head	34.05 ± 7.79 *	23.49 ± 5.14
Iliopsoas	5 ± 0.76 *	51.38 ± 5.32
Rectus femoris	5.67 ± 0.58	10.67 ± 2.52
Semitendinosus	38.33 ± 2.52 *	51.67 ± 4.04

Note: * represents a significant difference between bounced jump (BJ) and alternate jump (AJ), *p* < 0.05.

## Data Availability

The data that support the findings of this study are available on reasonable request from the corresponding author. The data are not publicly available due to privacy or ethical restrictions.

## Abbreviations

**Acronyms**	**Meaning**
ACL	Anterior cruciate ligament
AJ	Alternate jump
BJ	Bounced jump
BW	Bodyweight
EMG	Electromyography
LASI	Left anterior superior iliac spine
LPP	Left iliac crest
LTROC	Left greater trochanter
MPJ	Metatarsophalangeal joint
MVC	Maximum voluntary isometric contraction
RASI	Right anterior superior iliac spine
RLK	Right outer knee
RM1	The first metatarsophalangeal joint
RM5	The fifth metatarsophalangeal joint
RMK	Right inner knee
ROM	Range of motion
RPP	Right iliac crest
RTOE	Right big toe
RTROC	Right greater trochanter
SACR	Sacroiliac joint center
SD	Standard deviation
SPM	Statistical parametric mapping
VALR	Vertical impact loading rate
VGRF	Vertical ground reaction force
